# Transparent DNA/RNA Co-extraction Workflow Protocol Suitable for Inhibitor-Rich Environmental Samples That Focuses on Complete DNA Removal for Transcriptomic Analyses

**DOI:** 10.3389/fmicb.2016.01588

**Published:** 2016-10-18

**Authors:** Natalie Y. N. Lim, Constance A. Roco, Åsa Frostegård

**Affiliations:** ^1^Department of Chemistry, Biotechnology and Food Science, Norwegian University of Life Sciences, AasNorway; ^2^Department of Microbiology, Cornell University, Ithaca, NYUSA

**Keywords:** RNA extraction, purification, genomic DNA removal, enzyme inhibition, environmental sample

## Abstract

Adequate comparisons of DNA and cDNA libraries from complex environments require methods for co-extraction of DNA and RNA due to the inherent heterogeneity of such samples, or risk bias caused by variations in lysis and extraction efficiencies. Still, there are few methods and kits allowing simultaneous extraction of DNA and RNA from the same sample, and the existing ones generally require optimization. The proprietary nature of kit components, however, makes modifications of individual steps in the manufacturer’s recommended procedure difficult. Surprisingly, enzymatic treatments are often performed before purification procedures are complete, which we have identified here as a major problem when seeking efficient genomic DNA removal from RNA extracts. Here, we tested several DNA/RNA co-extraction commercial kits on inhibitor-rich soils, and compared them to a commonly used phenol-chloroform co-extraction method. Since none of the kits/methods co-extracted high-quality nucleic acid material, we optimized the extraction workflow by introducing small but important improvements. In particular, we illustrate the need for extensive purification prior to all enzymatic procedures, with special focus on the DNase digestion step in RNA extraction. These adjustments led to the removal of enzymatic inhibition in RNA extracts and made it possible to reduce genomic DNA to below detectable levels as determined by quantitative PCR. Notably, we confirmed that DNase digestion may not be uniform in replicate extraction reactions, thus the analysis of “representative samples” is insufficient. The modular nature of our workflow protocol allows optimization of individual steps. It also increases focus on additional purification procedures prior to enzymatic processes, in particular DNases, yielding genomic DNA-free RNA extracts suitable for metatranscriptomic analysis.

## Introduction

With the advent of the meta-omics era, it has become increasingly commonplace to aim for metagenomic/metatranscriptomic analyses of environmental samples. Despite advances in the sequencing front, upstream methods required to obtain the high quality DNA and RNA needed for these analyses have fallen behind and there is often a need to optimize existing methods when applying them to a new sample type. The choice of extraction method affects the ensuing purity and yield of nucleic acid material, which in turn affects subsequent downstream processes. This calls for rapid and simple extraction and/or purification methods that yield high quality and quantities of nucleic acids. However, this is but a pipe dream in many cases, due to the presence of “inhibitory compounds.” These well-known, yet poorly understood compounds are ubiquitous to most environments. They are abundant in most soils and are often classified under the blanket term of “humic and fulvic compounds, and/or polyphenolic compounds” (Tebbe and Vahjen, 1993; Krsek and Wellington, 1999; Hirsch et al., 2010; Mettel et al., 2010), yet there is little certainty that this is an accurate enough description of all enzyme-influencing compounds present in soil. Additionally, although it is known that inhibitors affect many DNA-transforming processes including hybridization, quantification and amplification (Tebbe and Vahjen, 1993; Bachoon et al., 2001; Zipper et al., 2003; Wang et al., 2012), many studies focus primarily on their effect on DNA polymerases (Abu Al-Soud and Rådström, 1998; Kermekchiev et al., 2009; Baar et al., 2011), disregarding the effect these same inhibitors may have on other enzymes performing other processes. Another complicating factor is that enzymes show various degrees of resistance to different inhibitors (Tebbe and Vahjen, 1993; Abu Al-Soud and Rådström, 1998; Baar et al., 2011). Thus, along with the development of new and efficient enzymes, there is a strong need for improved purification strategies.

Presently available methods can be divided into two: those that co-extract both DNA and RNA from single reactions, and those that extract DNA and RNA from separate reactions. While extracting nucleic acids separately is markedly simpler, with a wider variety of highly optimized kits and methods available, single reaction DNA/RNA co-extractions offer the benefit of more comparable data, especially from highly heterogeneous samples such as soils. This has spawned a multitude of novel methods and kits from independent researchers (Purdy et al., 1996; Griffiths et al., 2000; Peršoh et al., 2008; Mettel et al., 2010; Lever et al., 2015) and large multinational companies alike, as well as many comparisons of such methods and kits (Krsek and Wellington, 1999; LaMontagne et al., 2002; Dineen et al., 2010; Mahmoudi et al., 2011; Vishnivetskaya et al., 2014). Despite extensive testing of both kit and non-kit based methods, no single method has been found to work for all environment types (Frostegård et al., 1999; Krsek and Wellington, 1999; LaMontagne et al., 2002; Vishnivetskaya et al., 2014), and the “best” method is often difficult to determine, where one kit or reagent may provide, for example, better replication or quantity, but at the detriment of quality (Krsek and Wellington, 1999; Mahmoudi et al., 2011; Cruaud et al., 2014; Vishnivetskaya et al., 2014). Furthermore, there are fewer studies based on metatranscriptomics compared to metagenomics, resulting in a disproportionate focus on DNA-based methods over RNA ones.

Metatranscriptomic analyses require high quality RNA that is free of inhibitors and genomic DNA (gDNA). The presence of inhibitors greatly affects RNA high throughput sequencing due to the relatively large quantities of RNA required. Unlike DNA-based analyses, where “diluting out the inhibitor effect” is always an option, metatranscriptomic analyses often require concentrating samples in order to achieve sufficient material for the sequencing process, thus further exacerbating the inhibitory effect. Even if we ignored any effect the inhibitory compounds may have on the RNA extraction and DNA removal process, this need to concentrate samples makes inhibitor removal an extremely important step in RNA analysis. Thus, there is a consistent necessity to optimize existing methods and/or kits to suit one’s needs. Although commercial kits have the potential to yield high quality nucleic acids, the proprietary nature of kit components make it difficult for optimization or up-scaling. Such changes to the extraction procedure or increased sample volumes may be necessary for samples with low biomass and/or activity, containing little mRNA, when metatranscriptomic analysis is sought after.

The present study aimed to identify and overcome key problematic steps during the co-extraction of high quality DNA and RNA from inhibitor-rich soil samples for the purposes of meta-omic analyses. The efficacy of commercially available nucleic acid extraction kits were tested, and the nucleic acid extracts’ yield and purity were compared to the extracts obtained using the method by Nicolaisen et al. (2008) that was used in a previously published paper investigating the same soils (Liu et al., 2010). Finding little benefit in using the extraction kits, we took lessons learnt from a different modular extraction method (Lever et al., 2015), and further optimized Nicolaisen et al. (2008) method in an iterative manner, starting with the types of beads used for cell lysis and the nucleic acid precipitant. Different purification kits were also compared by examining the efficiencies of nucleic acid targeting enzymes (polymerases, DNases and reverse transcriptases) used on crude total nucleic acids (TNA) extracted by the aforementioned optimized method. Special attention was paid to the removal of gDNA from RNA samples. This step is often incorrectly assessed, despite being a potential source of major bias in downstream mRNA analyses. The proposed protocol, which is an optimization of existing phenol-chloroform based procedures, with additional purification at critical points, proved to yield nucleic acids suitable for metagenomic and metatranscriptomic analyses when tested on soils with high levels of inhibitors. The new method and workflow are transparent, which allows optimizations (as necessary) at various steps in the procedure.

## Materials and Methods

### Soils

Three agricultural soils, chosen because of their extraction difficulty with commercial kits and non-kit methods (Liu et al., 2010), were used to determine the quality of DNA and RNA from co-extraction reactions. Soils FL (pH 3.65) and FH (pH 7.39) are high organic content peat soils (40–45% soil organic C, 2% organic N) (Liu et al., 2010) from a long-term field experimental site in Fjaler in western Norway (61°17′42′′, 5°03′03′′). FL is the original un-limed soil, and FH was limed in 1978 with 800 m^3^ of shell sand per hectare of soil (Sognnes et al., 2006). Soil Å (pH 5.5) is a high clay-content soil (39% sand, 40% silt, 21% clay, 3% soil organic C, 0.22% organic N) from a grassland site in Ås in southeast Norway (59°39′44′′, 10°45′50′′). All soils were immediately transported to the laboratory, sieved (4.5 mm) upon arrival, then stored in sealed plastic bags at 4°C. All pH values were measured in 0.01 M CaCl_2_ (1:5 (ww to volume) soil to CaCl_2_ solution) immediately prior to using the soil. Soils FH and FL were used in the testing of all kits and methods, and soil Å was only used as a comparison for kits/methods that showed at least some success with the other two soils.

### Soil Treatment

In the present study we targeted denitrification gene transcripts to evaluate methods for DNA/mRNA isolation. Several successive experiments were performed where different extraction kits/methods were tested (see below). Using field-fresh soil for each of these would introduce undesired variation, due to seasonal differences in the soil. Instead, to achieve the best possible comparison of extraction methods, all soils used in this study were sampled at the same time and kept at 4°C until use (2–6 months after arrival).

A small amount of a natural C source was added, to standardize the conditions and to secure that the organisms would have enough energy to induce transcription of the targeted denitrification genes (Liu et al., 2010). Soils FH and FL were revitalized from cold storage by addition of 5 mg dried, powdered clover g^-1^ soil wet weight (ww), amended with 8–11 mM nitrate (in soil moisture), then incubated at 15°C for 72 h. Soil Å was used in a separate experiment (C. A. Roco, unpublished data) and was exposed to different lengths of oxic and anoxic periods over 4 weeks in glass vials incubated at 15°C. During this incubation, clover (1 mg g^-1^ soil, dry weight (dw)) and nitrate (0.065–0.65 μmol g^-1^ soil, dw) was added every 2–5 days (for a total of 11 times) to maintain microbial activity.

At the end of the 72 h (FH and FL) or 4 weeks (Å) incubation, the soils were transferred to air-tight glass vials and sealed with butyl-rubber septa and aluminum crimps, then made anoxic by six cycles of gas evacuation and helium filling (Liu et al., 2010). These vials were incubated anoxically to stimulate the production of denitrification gene transcripts. Gases (CO_2_, O_2_, NO, N_2_O, and N_2_) produced in the headspace were measured every 3 h with a GC and NO analyzer (Molstad et al., 2007), and used to guide soil sampling for denitrification genes – reduction of N_2_O gas to N_2_ gas was taken as an indicator for nitrous oxide reductase gene (*nosZ*) transcription. For each sample, one vial was opened and the soil within was snap frozen in liquid nitrogen then stored at -80°C until nucleic acid extraction.

### Kit and Non-kit Nucleic Acid Extraction

**FIGURE 1:**
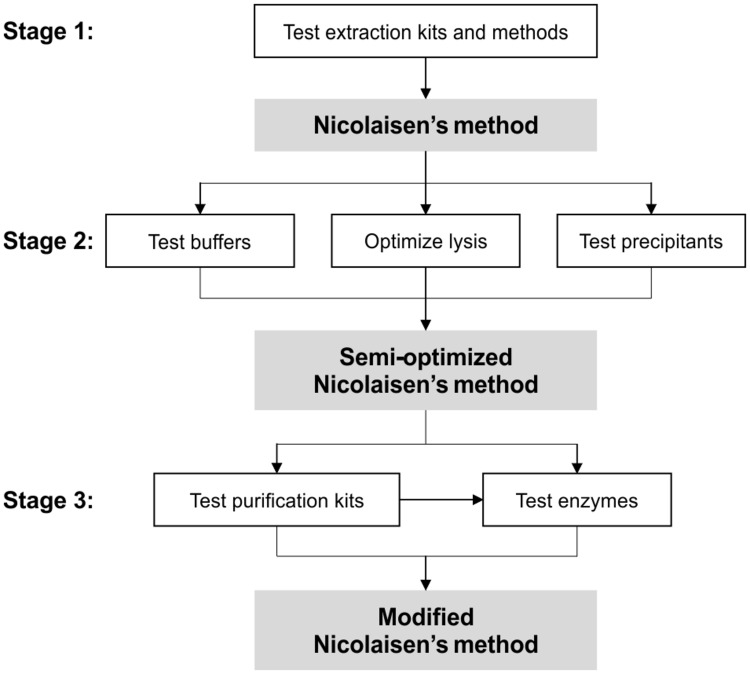
**Schematic diagram of the optimization process.** In Stage 1 of the process, various extraction kits and Nicolaisen’s method (as listed in **Table 1**) was tested on soils FH and FL (see text for soil descriptions). In Stage 2, various extraction buffers, lysis conditions, and nucleic acid precipitants were tested using Nicolaisen’s method as the base, creating a new “semi-optimized Nicolaisen’s method.” In the final Stage 3, DNases/reverse transcriptases and purification kits were tested concurrently for their ability to completely remove genomic DNA, and was briefly tested in combination. The end result is the “Modified Nicolaisen’s method,” which is based on the workflow as outlined in **Figure 2**.

**Figure 1** presents a schematic diagram of the different key steps examined to obtain an optimized protocol for co-extraction of DNA and RNA from soil. Our criteria for the successful application of a kit or method was the ability to obtain high quality DNA and RNA (both rRNA and mRNA) from our samples. Quality was assessed as follows: (1) DNA extracts should be amplifiable with little or no inhibition, as judged by successful PCR amplification and comparable qPCR efficiency to plasmid standards; and (2) RNA extracts must be free of gDNA (as determined by qPCR, see below), and should yield positive results when reverse transcribed and assessed with qPCR. Three DNA- and three TNA extraction kits were tested for their ability to extract nucleic acids that are suitable for downstream processes, according to manufacturer’s instructions (**Table 1**). In the present paper, the RNA PowerSoil kits are considered one kit because the DNA Accessory Kit (AK) cannot be used separately. Where applicable, lysis was achieved by bead-beating as described below.

**Table 1:** List of extraction and purification kits tested in this study^a^.

Use	Target	Kit name	Abbreviation	Company
Extraction	DNA	PowerLyzer PowerSoil DNA Isolation Kit	PL	MO BIO Laboratories
Extraction	DNA	FastDNA SPIN Kit for Soil	FDS	MP Biomedicals
Extraction	DNA	ZR Soil Microbe DNA MiniPrep	SM	Zymo Research
Extraction	DNA/RNA	MasterPure RNA Purification Kit^b^	MP	Epicentre Biotechnologies
Extraction	DNA/RNA	PowerMicrobiome RNA Isolation Kit	PM	MO BIO Laboratories
Extraction	RNA	RNA PowerSoil Total RNA Isolation Kit	PS	MO BIO Laboratories
Extraction	DNA	RNA PowerSoil DNA Elution Accessory Kit (used in conjunction with the above RNA kit)	AK	MO BIO Laboratories
Purification	DNA	E.Z.N.A. Cycle Pure Kit	CP	Omega Bio-Tek
Purification	DNA	MinElute Reaction Cleanup Kit	MRC	QIAGEN
Purification	DNA	Genomic DNA Clean & Concentrator	gDCC	Zymo Research
Purification	RNA	RNeasy Mini Kit	RM	QIAGEN
Purification	RNA	RNA Clean & Concentrator – 5	RCC	Zymo Research
Purification	DNA/RNA	*OneStep* PCR Inhibitor Removal Kit	OPIR	Zymo Research

*^a^The purification kits were tested in combination with the modified method described in this paper.*

*^b^The lysate was obtained using the phenol-chloroform extraction as detailed previously (Nicolaisen et al., 2008).*

The PowerLyzer DNA (PL), FastDNA SPIN (FDS), and ZR Soil (SM) kits were used as benchmark DNA extractions because of their previous success in our laboratory with soil FH and in the literature in extracting DNA from soil and other environmental samples (Mahmoudi et al., 2011; Vishnivetskaya et al., 2014; Wesolowska-Andersen et al., 2014; Young et al., 2014). The rest of the kits were selected according to the manufacturer’s claim that they are able to co-extract DNA and RNA fractions from the same soil sample. The kits were compared to the phenol-chloroform extraction method as modified by Nicolaisen et al. (2008), referred to here as the Nicolaisen’s method, which is based on the extraction procedure by Griffiths et al. (2000).

The lysis step of Nicolaisen’s method was optimized by testing different lysis options (FastPrep-24 Instrument vs. vortex), lysis beads type (garnet vs. glass), one size (garnet: 0.15 mm; glass: 0.10-0.11 mm) vs. multiple bead sizes (garnet beads: 0.15 and 0.7 mm; glass beads: 0.10–0.11, 1.0, and 2.5–3.5 mm), and the number of cycles of lysis (once, twice, or thrice). Different buffers for the lysis of bacteria were also tested: CTAB (hexadecyltrimethylammonium bromide) buffer (pH 5.7 and 8.0, and 120 mM or 250 mM ionic strength) with 1% (w/v) polyvinylpolypyrrolidone (PVPP); GES (guanidinium thiocyanate-EDTA-sarcosyl) buffer (pH 4); and phenol (pH 4 or 8) (Supplementary Table S1). Additionally, we tested the effectiveness of 30% polyethylene glycol (PEG) 6000 (following Nicolaisen’s method) and isopropanol as nucleic acid precipitants. The results are described in Supplementary Material, pp. 1–2 and Supplementary Figures S1–S5.

### Purification Kits

**FIGURE 2:**
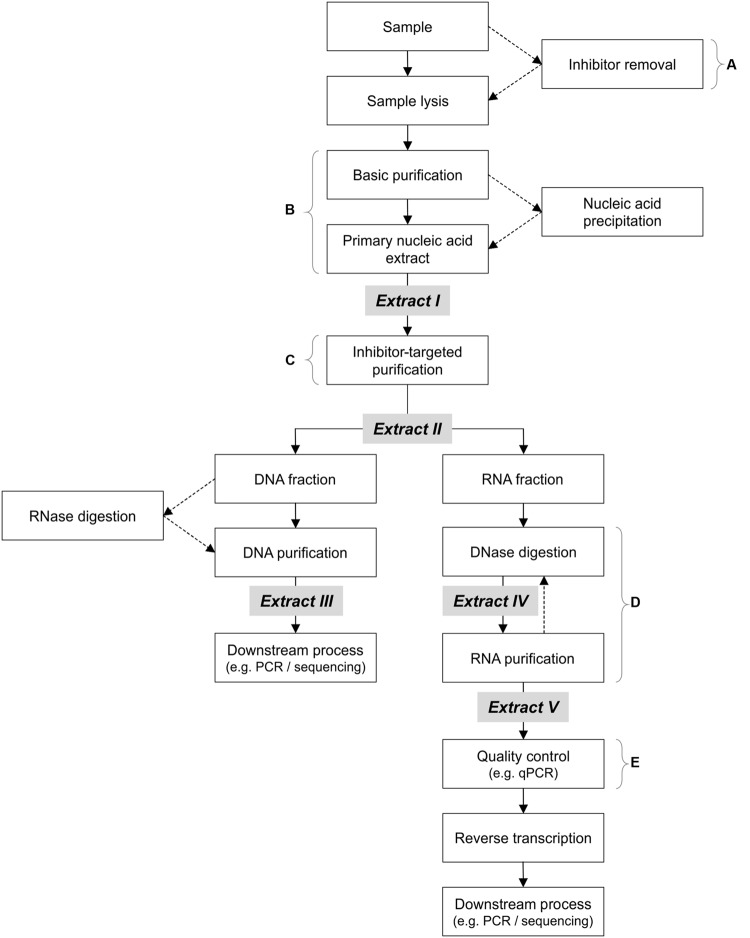
**Suggested DNA/RNA co-extraction workflow for environmental samples, with stronger emphasis on thorough purification prior to all enzymatic steps (including DNase digestion).** Optional steps are indicated by dotted arrows. Note that RNase digestion (between Extracts II and III) may be necessary for better results downstream, but may be omitted as a separate step (in the current study, RNase is present in the qPCR mix). **(A)** Pre-lysis inhibitor removal is only advisable if quick methods are used, or if mRNA is not the target molecule (lengthy inhibitor removal procedures compromise RNA integrity). **(B)** Various methods may be used, such as phenol/chloroform procedures or nucleic acid precipitation. **(C)** This purification step should target the removal of enzymatic-inhibitors (e.g., humic/fulvic acids and polyphenolics). **(D)** Purification of partially digested RNA extracts with residual genomic DNA aids in the removal of enduring inhibitors, prior to further digestion. **(E)** Stringent and well-documented quality control via rigorous and sensitive detection (preferably quantitative methods) is necessary to detect residual amplifiable gDNA **prior** to reverse transcription.

In the following, the term “primary” when used to describe nucleic acids refers to the resuspended or eluted nucleic acids obtained from the extraction procedure or kit, and is equivalent to “Extract I” in **Figure 2**. In addition to the purification steps already included in the above extraction methods and kits to obtain the primary extract, purification kits (listed in **Table 1**) were tested in various combinations on the primary extracts: MinElute Reaction Cleanup Kit (MRC), RNeasy Mini Kit (RM) (both from QIAGEN), E.Z.N.A. Cycle Pure Kit (CP) (Omega Bio-Tek), Genomic DNA Clean & Concentrator (gDCC), RNA Clean & Concentrator-5 (RCC) and *OneStep* PCR Inhibitor Removal Kit (OPIR) (all from Zymo Research).

### DNase Digestion of Total RNA

Based on our previous experience (Liu et al., 2010), residual gDNA is often leftover after DNase treatment of RNA fractions, making this step a major bottleneck, especially for inhibitor-rich soil samples. The following DNases were tested for their ability to remove amplifiable DNA from TNA samples: DNase I (Sigma), RNase-Free DNase Set (QIAGEN), RNase-Free DNase I (Epicentre Biotechnologies) and TURBO DNA-*free* DNase Kit (Ambion, Life Technologies). All DNases were used according to manufacturers’ instructions, with the exception of incubation time, which we varied from 15 min to 2 h. The efficiency of each DNase treatment was determined by comparing the purified DNA fractions (Extract III in **Figure 2**) with the non-reverse transcribed RNA (Extract V in **Figure 2**), via quantitative PCR (qPCR) amplification of the 16S rRNA or the *nosZ* genes (details below).

### Reverse Transcriptases

Several reverse transcriptases were compared using RNA extracts obtained from soils FL and FH during the iterative method optimization. The purpose was to ensure successful cDNA synthesis in extraction replicates from inhibitor-rich soils. Because trials with RNA extracts from Nicolaisen’s method and the extraction kits were not able to yield cDNA (see Comparison of Methods for Nucleic Acid Extraction, Supplementary Data section “The Effectiveness of Dedicated Nucleic Acid Extraction Kits,” and an earlier study Liu et al., 2010), the assessment focused on the presence (but not quantity) of detectable *nosZ* cDNA in the absence of gDNA. Reverse transcriptase efficiency was not assessed in this study. The following reverse transcriptases were tested according to manufacturers’ instructions: High Capacity RNA-to-cDNA Master Mix (Applied Biosystems), SuperScript VILO MasterMix (Invitrogen), PrimeScript RT Reagent Kit (Takara Bio), and Maxima Reverse Transcriptase (Thermo Scientific). Random hexamer primers and dNTPs (provided by the respective manufacturers, either bought separately or provided in the kit) were used with all reverse transcriptases. To improve the rate of successful *nosZ* transcript reverse transcription (present in low quantities in the samples compared to 16S rRNA), the maximum volume of RNA template (8–10 μL, corresponding to 150–200 ng RNA) was used in each reaction. Due to the comparatively low quantities of RNA in the extracts (compared to pure culture RNA extractions), the quantity of RNA in these volumes never exceeded the manufacturers’ recommended maximum quantity of RNA template (ranging from 500 ng to 5 μg total RNA). Additionally, the differing template quantities/volumes used in this study did not affect the failure or success of cDNA synthesis, as determined by the absence or presence of amplifiable *nosZ* cDNA (see Test of DNases and Reverse Transcriptases).

### Optimized Non-kit Extraction Method That Mitigates Inhibitor Effect

Based on the results from the above tests (as described in Supplementary Material, pp. 1–2 and Supplementary Figures S1–S5), some additions and modifications were made based on several widely used phenol-chloroform extraction methods, including Nicolaisen’s method (Griffiths et al., 2000; Nicolaisen et al., 2008; Mettel et al., 2010). **Figure 2** depicts our suggested workflow protocol, and is the basis for our method. Briefly describing the method, 0.2–0.25 g of soil was lysed by bead-beating in 2 mL screw-capped microcentrifuge tubes containing glass beads, CTAB extraction buffer (with 1% w/v PVPP), and phenol-chloroform-isoamyl alcohol (25:24:1), and the nucleic acids were washed with ethanol then precipitated. The following are the differences to Nicolaisen’s method: (i) Three sizes of glass beads were used for lysis (0.10–0.11, 1.0, and 2.5–3.5 mm); (ii) the samples were lysed in a FastPrep-24 Instrument by two cycles at 6.0 m s^-1^ for 45 s, with intermittent cooling between each cycle to prevent overheating of the samples and instrument; (iii) after removing residual phenol with chloroform, up to 500 μL of the aqueous phase was transferred; (iv) the nucleic acids (both DNA and RNA) were precipitated with 0.2 volumes of 3 M sodium acetate (buffered to pH 5.2 with glacial acetic acid) and an equal volume of isopropanol, then continuously inverted for 2 min at room temperature; and (v) the ethanol-washed TNA pellet was dried in a SpeedVac Concentrator then resuspended in DEPC-treated nuclease-free water.

After this primary extraction, and before any further enzymatic downstream treatment, the resuspended TNA (Extract I in **Figure 2**) was purified with the OPIR kit, according to manufacturer’s instructions. Extract II (**Figure 2**) was then divided in two fractions, one for DNA and one for RNA. To ensure maximum removal of inhibitory compounds, the DNA fraction was further purified with the gDCC kit. For the RNA fraction, gDNA was removed with the TURBO DNase kit, before purification with the RCC kit. If residual gDNA was detected in the eluate (via qPCR using primers targeting the 16S rRNA or *nosZ* genes), a second round of DNase digestion and purification with the RCC kit was performed (but without the OPIR kit prior to digestion). Additional use of OPIR prior to the second digestion did not improve RNA purity, but instead resulted in the loss of material (data not shown). The qPCR-certified gDNA-free RNA was then reverse transcribed to cDNA with random hexamers using the Maxima Reverse Transcriptase, both according to manufacturer’s instructions. All resulting nucleic acids (DNA, non-reverse transcribed RNA, and cDNA) were quantified after extraction and/or purification (see below), then stored at -80°C until use. This procedure of ‘purification before enzymatic processes’ was also used on primary extracts from the most effective extraction kit, RNA Powersoil kit (PS), to ensure high quality RNA for sequencing (see Results section).

### Analysis of Nucleic Acid Quality and Quantity

Extracts II, III, IV, and V (the primary TNA, purified DNA, the DNase-treated RNA, and purified RNA fractions, respectively; see **Figure 2**) were quantified by spectrofluorometry using the Qubit dsDNA BR Assay Kit and Qubit RNA BR Assay Kit (Qubit Fluorometer, Invitrogen, Life Technologies). Spectrophotometric analysis (NanoDrop Spectrophotometer, NanoDrop Technologies, Thermo Fisher Scientific) was used for preliminary evaluation of nucleic acid quality, via the assessment of the absorbance ratios *A*_260/230_ and *A*_260/280_. As is common practice, *A*_260/230_ absorbance ratios nearing 2.0 were regarded as contaminated with humic substances, whereas ratios below 1.5 were regarded as failure to extract nucleic acids (Cullen and Hirsch, 1998; Krsek and Wellington, 1999; LaMontagne et al., 2002; Peršoh et al., 2008; Mahmoudi et al., 2011). However, due to the high quantities of humic compounds present in soils FL and FH, we only regarded it as failed nucleic acid extraction if the ratio remained under 1.5 after additional clean-up with dedicated purification kits. Protein contamination was indicated by the *A*_260/280_ ratio, where samples with ratios between 1.7 and 2.0 were considered usable, while purified extraction reactions with ratios < 1.7 were discarded. Estimation of humic content by color (Dineen et al., 2010) was not used in this study, since low amounts of humic substances may be undetectable visually (Bachoon et al., 2001). Additionally, where applicable, gel visualization was used to quickly assess the extent of DNA shearing and/or the presence of rRNA (note that rRNA presence/absence was always further confirmed by PCR/qPCR following reverse transcription). For reasons of simplicity, in this paper the term “usable nucleic acids” refers to nucleic acids of sufficient enough quality to be used in further experiments, i.e., downstream processes such as qPCR were not inhibited or inversely affected by co-extracted inhibitory compounds.

### Verification of Inhibitor and gDNA Absence

To confirm amplifiability of extracted DNA and synthesized cDNA, and the complete digestion of gDNA in RNA samples, the presence of the 16S rRNA, *narG* and *nosZ* genes were assessed via PCR and qPCR. For both PCR and qPCR, DNA samples were diluted to between 1:10 and 1:50 of the original extract, which translated to 1–10 ng of DNA per reaction. All cDNA and RNA samples (DNase-digested) were used without dilution. For PCR, each 25 μL amplification reaction contained 1 μL of template, 0.4 μM of each primer, 0.125 U of *TaKaRa* Taq (Takara Bio), 400 μM of each dNTP and 2.5 μL of 10X PCR Buffer. The primers used were: 27F and 518R for the 16S rRNA gene (Weisburg et al., 1991; Muyzer et al., 1993), 1960f and 2650r for the *narG* gene (Philippot et al., 2002), and Z-F and 1622R for the *nosZ* gene (Kloos et al., 2001; Throbäck et al., 2004). The optimized thermal cycling conditions were 95°C for 5 min, 30–35 cycles of 95°C for 30 s, *x* for 45 s, 72°C for 30 s, and a final extension of 72°C for 7 min, where *x* = 54°C (16S rRNA gene), or 60°C (*narG* and *nosZ* gene). For qPCR the StepOnePlus Real-Time PCR System (Applied Biosystems) was used. All samples were amplified in simultaneous reactions to compare the DNase digestion and reverse transcription efficiency. Each 20 μL reaction contained SYBR *Premix Ex Taq* II (Tli RNaseH Plus; Takara Bio) used according to manufacturer’s instructions, and included 0.4 μM of each primer and 2 μL of template. The qPCR cycling conditions for all primer sets were the same as above, with the following exceptions: an additional 20 s at 82°C at the end of each cycle to measure the fluorescent signal, thereby reducing background signals from primer dimers and unspecific PCR products; the extension time for the primers targeting the *nosZ* gene was prolonged to 60 s; a final melting curve analysis from 60 to 95°C was performed to determine the specificity of amplicons, in lieu of the final extension step; and the amplification reactions were performed for 40 cycles. The detection limit of each qPCR run was five copies per microliter of reaction, which ranged from 4 × 10^2^ to 4 × 10^5^ copies g^-1^ soil (ww).

The raw qPCR fluorescence data was imported into the LinRegPCR program (Ruijter et al., 2009). Unlike commonly reported efficiencies that are calculated by employing the use of serial diluted standards and the construction of calibration plots, LinRegPCR uses the exponential portion of the fluorescence signal curve of each well to determine individual well efficiencies by calculating the deviation from a perfect “one copy to two copies” amplification after each cycle. Efficiencies calculated with standard curves assume equal amplification efficiencies in all calibration and biological samples, and cannot be used objectively to determine the degree of amplification inhibition in biological samples. To overcome this, qPCR curve analysis methods such as LinRegPCR, as used above, have to be used (Ruijter et al., 2013). This allows for more reliable qPCR efficiency determinations that are independent of potential standard-sample variations, including differences in inhibitor content. Moreover, humic substances have been found to inhibit commonly used double-stranded DNA (dsDNA) binding fluorescence dyes, making it doubly important to check individual sample amplification efficiencies (Sidstedt et al., 2015).

### Additional Nucleic Acid Quality Control and Sequencing

Multiple samples of DNA and RNA extracted from all three soils using our revised extraction method, and PS kit-extracted (and further purified as described in the simplified extraction method) soil Å RNA extracts, were sent for metagenomic and metatranscriptomic sequencing at The Roy J. Carver Biotechnology Center (CBC)/W. M. Keck Center for Comparative and Functional Genomics at the University of Illinois at Urbana-Champaign, using HiSeq 2500 technology. Prior to shipping on liquid nitrogen vapor (Cryoport), we confirmed that all nucleic acids were of high quality (DNA or gDNA-free RNA as verified by qPCR). Independent verification of the RNA quality, including confirmation of the absence of gDNA, was also performed at the CBC. A sample of the sequenced reads from soil FH and FL were trimmed for adaptors and quality using Trimmomatic (MINLEN: 70, TRAILING: 15) (Bolger et al., 2014). The trimmed sequences were uploaded to MG-RAST and annotated (Meyer et al., 2008). Annotated FH and FL soil sequences are available online on the MG-RAST database (project ID 14446, project name “Fjaler_HiSeq”).

## Results

### Comparison of Methods for Nucleic Acid Extraction

No single dedicated nucleic acid extraction kit was applicable to all soils. The kits that managed to obtain both DNA and RNA (kits MP, PM and PS+AK) are compared to the unmodified Nicolaisen’s method in **Table 2**. For a comparison of all kits tested, see Supplementary Table S2 and explanatory text in Supplementary Material, p. 1. As seen, PS was the most successful kit, obtaining gDNA-free RNA in two of the three soils. The PS kit utilizes nucleic acid-specific elution buffers to preferentially elute DNA or RNA from the nucleic acid binding column. However, as per manufacturer’s strict instructions, neither centrifugal (positive) nor vacuum (negative) pressure could be applied to the columns (supplied in the kit), and the gravitational drip process took over 4 h (and up to 8 h) per sample to complete for FL and Å soils, due to clogging of the column. Despite the long procedure at room temperature, preliminary trials with the PS kit (without the AK kit) produced promising results, yielding 6.71 ± 1.01 μg RNA g^-1^ soil (ww) and amplifiable cDNA (16S rRNA) in the absence of amplifiable gDNA. The long extraction time required at room temperature may potentially compromise the quality and quantity of extracted mRNA, which puts any absence or low mRNA copy numbers in doubt. The only available option provided by the manufacturer was the application of positive pressure to the top of the column. Unfortunately, the outcome varied between soil types Å and FL: High quality rRNA and mRNA was obtained from soil Å, although a supplementary two rounds of ‘purification-digestion-purification’ was required (i.e., RNA purification was performed after each digestion). In contrast, for soil FL, positive pressure application co-extracted such large quantities of inhibitory compounds that both the extracted DNA (eluted with the AK kit) and RNA remained brown (suggesting a high content of organic compounds) and was unusable in downstream processes in spite of attempted clean-up with additional purification kits. Moreover, the extracts were not reliably quantifiable prior to further purification (NanoDrop and Qubit readings returned “error” and “out of range” messages, respectively). NanoDrop quality assessments revealed highly variable *A*_260/280_ ratio ranges that failed to improve with additional purification: 1.41–1.58 for the DNA eluate and 1.34–1.79 for the RNA eluate (see also Supplementary Table S2). Tellingly, the DNA and reverse transcribed RNA could not be amplified (fluorescence signal did not pass threshold after ≥35 cycles in the qPCR using primers targeting the 16S rRNA gene). The PS kit therefore did not provide sufficient quality of nucleic acids from soil FL because of the long extraction time required at room temperature and the inability to speed up the process with positive pressure application.

**Table 2:** Comparison of DNA and RNA co-extraction methods and kits, tested on soils FH (high pH peat, pH 7.39), FL (low pH peat, pH 3.65), and Å (low pH clay soil, pH 5.5).

Method/Kit	Nicolaisen’s method^a^			MP^b^		PM^b^		PS + AK^b^			Optimized method		
TNA purification prior to digest^c^	-			+		-		+			+		
Soils tested	FH	FL	Å	FH	FL	FH	FL	FH	FL	Å	FH	FL	Å
Amplifiable DNA^d^	+	+	+	+	+	+	+	+	±^f^	+	+	+	+
Complete removal of DNA after 1st digestion^d,e^	+	-	-	+	-	+	-	+	-	-	+	+	-
Complete removal of DNA after 2nd digestion^d,e^	+	-	-	+	±^f^	+	-	+	-	+	+	+	+
cDNA synthesis	+	NT	NT	+	±^f^	+	NT	+	NT	+	+	+	+

*^a^Method from Nicolaisen et al. (2008).*

*^b^See **Table 1** for list of kit abbreviations.*

*^c^TNA purification with the OPIR kit.*

*^d^See text for details on DNA amplification and removal assessment.*

*^e^DNA was digested with TURBO DNase, and RCC kit was used for purification after each digestion.*

*^f^Results from replicates varied, likely due to the presence of inhibitory compounds.*

*gDNA, genomic DNA; NT, not tested because of residual gDNA.*

### Purification Kits and Enzymatic Inhibition

In the final stage of optimization (**Figure 1**), various purification kits (listed in **Table 1**) were tested on FH and FL extracts from the best extraction kits (listed in **Table 2**) and our optimized version of Nicolaisen’s method (utilizing the most optimally tested buffer and precipitant as stated in the Supplementary Material, pp. 1–2). Regardless of method or kit used for the extraction, the DNA yielded from both FH and FL in Extract I (**Figure 2**) was amplifiable, but the results were variable in consistency and strength (strong and consistent amplification was defined by the presence of equally bright amplicons on agarose gels, see Supplementary Figure S1). Due to the inhibitor-rich nature of the soils tested, we found that nucleic acid purification kits were always necessary to secure high quality, fully uninhibited material for downstream processes such as PCR amplification.

These further purification steps, regardless of the purification kit used, greatly improved the purity of DNA extracts. For example, purification of FL extracts with gDCC improved the *A*_260/280_ ratio from 1.59 ± 0.05 to 1.81 ± 0.09, and the *A*_260/230_ ratio from 1.17 ± 0.07 to 1.65 ± 0.04. Eluates from these DNA purification kits were always amplifiable: Amplification of these purified DNA extracts resulted in brighter and more consistent amplicon bands (on agarose gel) when the same quantity of pre-purification DNA was used, independent of primers used (Supplementary Figure S1). This indicated that the inhibitory compounds interfering with the PCR amplification of the TNA (Extract I, **Figure 2**) were removed by purification with DNA clean-up kits (note that step C in **Figure 2** had not yet been included during this early purification kit testing).

For RNA, on the other hand, the quality of the extracts varied, as seen from differences in residual gDNA for soils FH and FL below. We were able to obtain gDNA-free RNA from soil FH (gDNA undetectable via qPCR analysis after 35 cycles), although DNase digestion was always required to remove the residual gDNA, regardless of kit or method used (including the PS kit, despite its preferential eluent system). These RNA extracts from soil FH were successfully reverse transcribed, as judged from the amplification of the resulting cDNA using qPCR (detected after ≤35 cycles). In contrast, RNA extracts from soil FL often contained qPCR-amplifiable gDNA (detected after ≤35 cycles) that was not removable even after repeated rounds of extended DNase digestion (1–2 h) and RNA clean-up kit purification (regardless of purification kit used). There was often residual gDNA in these primary extracts even after a second digestion or, in cases where gDNA was completely digested (in the qPCR), the RNA in the sample was no longer detectable (undetectable after ≥35 cycles, after reverse transcription followed by qPCR).

During the first two stages of optimization (**Figure 1**), we observed that enzymatic issues in the RNA fraction (e.g., incomplete DNase digestion as described above) coincided with Taq polymerase inhibition in the DNA fraction (polymerase inhibition is described above and in Supplementary Figures S1 and S4), suggesting that the same inhibitors associated with Taq polymerase activity could be the main reason behind the interference with other enzymes (i.e., DNase and reverse transcriptase). Thus in Stage 3 of optimization (**Figure 1**), we used the OPIR kit, a TNA purification kit that specializes in inhibitor removal, on the primary TNA Extract I (**Figure 2**) prior to any enzymatic process (including DNase digestion). In addition to improved DNA quality, we observed little loss of nucleic acid material. For example, purification of 3–4 μg of DNA g^-1^ soil (ww) resulted in 2.5–3.5 μg using OPIR (compared to 2–2.3 μg using gDCC), and the Extract II (**Figure 2**) DNA was as equally amplifiable as Extract III (**Figure 2**) DNA purified with dedicated DNA purification kits, confirming the removal of Taq polymerase inhibitors. The improved TNA quality was also observed by enhanced DNase digestion. A single, non-extended digestion using the TURBO DNase kit (see below), performed according to manufacturer’s instructions, reduced the quantity of residual gDNA in the digested RNA Extracts V (**Figure 2**) from FH and FL soils to below the limit of PCR and qPCR detection (conservatively estimated to 2 copies μL^-1^ reaction; in this case corresponding to 1.6 × 10^4^ 16S rRNA gene copies g^-1^ soil, ww).

Thus, we concluded that using the OPIR kit prior to a DNA or RNA purification kit was the best option for obtaining high quality DNA or RNA extracts, respectively. With the addition of the OPIR kit, we did not observe any difference in the quality of DNA or RNA yielded by any of the purification kits tested, so the choice of DNA and RNA purification kit used in subsequent extractions was decided by load capacity and cost per reaction. For our purposes, the OPIR, gDCC, and RCC kits satisfied these criteria and were used on the DNA and RNA extracts sent for metagenomic and metatranscriptomic analysis, respectively.

### Test of DNases and Reverse Transcriptases

In the second part of Stage 3 optimization (**Figure 1**), OPIR kit purified, inhibitor-free extracts from all three soil types were used to test different DNases (Extract II) and reverse transcriptases (Extract V). Of the DNases tested, TURBO DNase was the most active at 2 Units μL^-1^ (as described in the respective product information sheets), and was also the most efficient at removing gDNA from samples even in the presence of low quantities of inhibitors (residual gDNA was undetectable with qPCR after ≥35 cycles when using TURBO DNase, compared to ≤35 cycles using the other DNases). Coupling this DNase with the OPIR kit made a potent combination for alleviating the inhibitory effect, thus digesting more gDNA in the TNA extracts.

**FIGURE 3:**
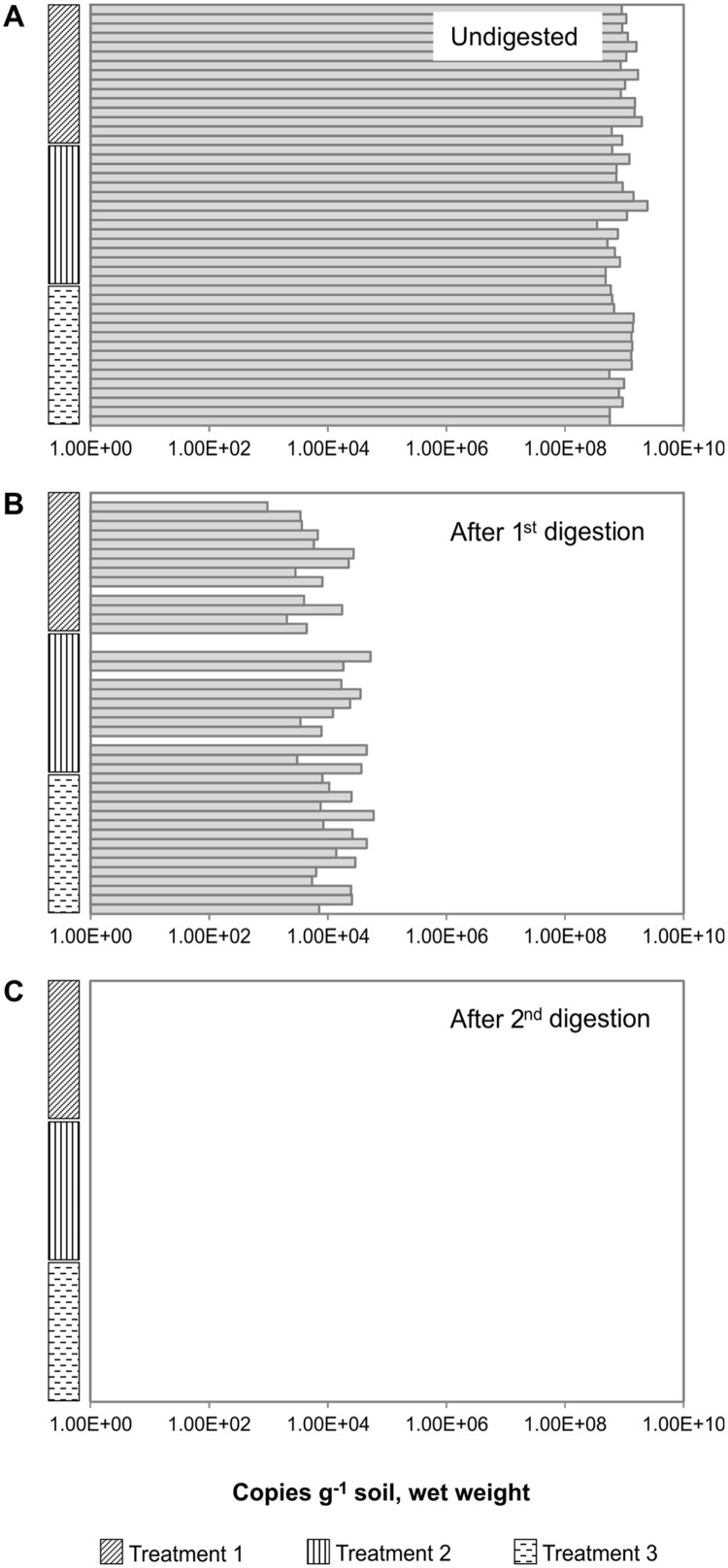
**Removal of gDNA by consecutive DNase digestions of total nucleic acids (TNA) extracted from 45 Å soil samples.** The soil had been exposed to different oxygen regimes (here called Treatments 1, 2, and 3), for details see section “Materials and Methods.” The soils were incubated anoxically to stimulate denitrification gene expression, and samples were taken at time intervals. TNA was extracted using the optimized and simplified method, and the *nosZ* was quantified by qPCR. **(A)** After extraction via the optimized method, all samples were tested for the presence of DNA. Neither the different oxygen regimes nor the stimulation of gene expression affected the number of *nosZ* genes in the gDNA from the different samples. **(B)** The first digest removed most amplifiable genomic DNA (gDNA) present. **(C)** The second DNase treatment removed amplifiable gDNA in all samples. There was no relationship between the starting DNA quantity and the success of complete gDNA removal (*R*^2^ = 0.0189). This highlights the importance of checking all RNA samples and not only representative samples, as there may be high variability among samples from the same source and extraction procedure.

To investigate the reproducibility of gDNA removal, we quantified the *nosZ* gene in TNA that was extracted from 45 soil Å samples and digested in two consecutive rounds (**Figure 3**). The soil had been exposed to different oxygen regimes, and incubated anoxically for different time periods (see Materials and Methods), but these treatments did not affect the copy numbers of *nosZ* in the gDNA content of the samples (**Figure 3A**). Although residual gDNA persisted in some samples from soil Å after the first DNase digestion (Extract IV), purification with an RNA purification kit (e.g., RCC) followed by a second DNase digestion often completely removed the remaining gDNA in Extract V (**Figure 3**). The first digestion ensured that any RNA clean-up kit used (in this case, RCC) did not become overloaded by the large quantities of extracted gDNA, which would result in the loss of RNA. Using qPCR on these RNA extracts, we showed that two rounds of DNase digestion reduced the number of *nosZ* gene copies to below the qPCR detection limit (conservatively estimated to 2 copies μL^-1^ reaction; in this case corresponding to 400 copies g^-1^ soil (ww)) for all samples (**Figure 3**). This is compared to a single DNase digestion, where only 6 of 45 samples had undetectable quantities of *nosZ* DNA, and the residual gDNA in the remaining samples was 0.002 ± 0.002% of the original. Although these percentage numbers are small, they translate to a residual gDNA of between 900 and 60 000 copies of *nosZ* genes g^-1^ soil (ww). Notably, the soil samples retained different quantities of residual gDNA in RNA fractions despite identical extraction procedures, as indicated by qPCR (**Figure 3**). This differed from the DNA fractions that contained equally amplifiable and relatively similar quantities of gDNA in replicate extractions (**Figure 3A**).

Using these high quality gDNA-free RNA extracts for reverse transcription, there was no observable difference in the cDNA synthesis success rate between the reverse transcriptases tested – *nosZ* cDNA was always undetectable in partially purified RNA, and consistently detectable in high-quality RNA, regardless of the reverse transcriptase used. In this study, Maxima Reverse Transcriptase was chosen for use with the optimized method because it had the highest capacity and was thus the least likely to be overloaded by the total RNA in each sample (5 μg total RNA). Thus, for the optimized method, we used a combination of the OPIR and RCC purification kits and TURBO DNase to obtain high-quality RNA extracts prior to cDNA synthesis with the Maxima Reverse Transcriptase.

### Optimized and Transparent Method for Non-kit Based Extraction

Using the results from the optimization of the lysis and precipitation steps of Nicolaisen’s method (see Supplementary Material, pp. 1–2 and Supplementary Figures S1–S5), we revised the method as described in the section “Materials and Methods.” We compared the revised method with the different extraction kits and the original Nicolaisen’s method, and observed no advantage to using extraction kits over our revised extraction method. In addition to the shorter average extraction time and quick precipitation, the quality and quantity of nucleic acids extracted using our revised method was equal, if not better, than all the other kits and methods tested. Using the above described combination of purification kits and DNase enzyme, we were able to obtain gDNA-free RNA fractions (Extract IV) in the FL and FH soils after only a 30-min DNase digestion. This is compared to persistent incomplete DNA digestion in soil FL despite extended DNase digestion times of up to 2 h using the unamended Nicolaisen’s method, proving that low digestion efficiencies are likely caused by the failure to remove inhibitory compounds. Using our optimized method, the average *A*_260/280_ and *A*_260/230_ ratios before purification (Extract I) were 1.84 and 1.66, respectively, and the crude extracted quantities were 50–150 μg DNA g^-1^ soil (ww) and 15–18 μg RNA g^-1^ soil (ww). Analysis by agarose gel electrophoresis revealed reproducible TNA extraction, with large quantities of extracted rRNA that was clearly visible on the gel (Supplementary Figure S2). After a 10- or 20-fold dilution (to attain the desired 1–10 ng of DNA per reaction, as specified in Materials and Methods), Extract I from all soils (FH, FL, and Å) was always at least weakly amplifiable with primers targeting the 16S rRNA gene (as visualized on agarose gels). Additional purification using the OPIR kit, followed by the gDCC and RCC kit for DNA and RNA, respectively, yielded nucleic acids that were always usable in downstream processes.

Using qPCR analysis and primers targeting the 16S rRNA and *nosZ* genes, we confirmed that the purified RNA fraction (Extract V) contained no detectable copies of gDNA. Average 16S rRNA copies were reduced from 1.08 × 10^11^ ± 3.32 × 10^10^ (soil FH) and 3.15 × 10^10^ ± 1.19 × 10^10^ (soil FL) copies g^-1^ soil (ww) to below the detection limit of qPCR (1.6 × 10^4^ copies g^-1^ soil, ww) in RNA extracts. The RNA extracts were also successfully reverse transcribed to cDNA, and qPCR-amplifiable with primers targeting the *nosZ* gene (3 × 10^6^ and 1 × 10^5^ copies g^-1^ soil, ww in soils FH and FL, respectively).

Analysis of the raw qPCR fluorescence data using LinRegPCR revealed similar efficiencies for both the samples and the purified plasmid standards (**Table 3**), confirming the absence of amplification or dsDNA-binding dye inhibitors in all our amplification reactions. Although these individual amplification efficiencies appear to be low, similar efficiencies seen in the standards indicate that the lower-than-expected efficiencies are likely an effect of poor primer-template matches or the formation of primer dimers affecting the amplification reaction, rather than the presence of inhibitory compounds. For comparison to other studies, the calibration plot-based method of efficiency calculation yields amplification efficiencies of 95.1 and 99.1% for the 16S rRNA and *nosZ* genes, respectively.

**Table 3:** Individual qPCR efficiencies based on LinRegPCR analysis of nucleic acids extracted from soils FH (high pH peat, pH 7.39) and FL (low pH peat, pH 3.65).

Target	Plasmid standard	FH	FL
16S rRNA gene	77.9 ± 3.44%	81.3 ± 3.18%	82.0 ± 3.49%
*nosZ* DNA	84.2 ± 5.05%	85.4 ± 3.97%	84.2 ± 3.36%
*nosZ* cDNA	Same as above	80.7 ± 2.51%	81.0 ± 2.86%

### Quality Assessment and Reproducibility of DNA and RNA Extracts

DNA and RNA (Extracts III and V) yielded by our simplified TNA extraction method (soils Å, FL, and FH) and RNA (Extract V) from the PS kit (soil Å) (all purified with OPIR/gDCC/RCC kits as described previously), were sent for Illumina HiSeq sequencing at the CBC. All samples were independently verified to be of high quality: RNA extracts were confirmed to be free of gDNA, and both DNA and RNA were successfully sequenced with HiSeq 2500 technology. The resulting sequences were annotated using MG-RAST, and a summary of the annotated data has been included in the Supplementary Table S3. Total Sequence and Clusters of Orthologous Groups (COG) breakdown profiles generated using MG-RAST were highly similar between replicate extractions for both soil FH and FL, indicating good co-extraction replication (Supplementary Figures S6 and S7). Further analysis of the sequences (normalized to Reads per Million, RPM) using bacterial housekeeping genes as a reference of comparison revealed good reproducibility of DNA and RNA extraction replicates (examples of data shown in **Table 4**). There was minor variation for some genes in the RNA duplicates (e.g., *fusA* in R5 and R6), but the reproducibility for the other genes points toward variability in *fusA* gene expression due to incubation conditions, rather than an extraction bias. Together, the sequenced metagenomes and metatranscriptomes give evidence to the reproducibility of DNA and RNA co-extraction using the optimized method.

**Table 4:** Example of DNA and RNA meta-ome sequencing reproducibility, based on Reads per Million (RPM) values from MG-RAST annotation of bacterial housekeeping genes, obtained from soils FH (high pH peat, pH 7.39) and FL (low pH peat, pH 3.65).

Gene	FH					FL				
	DNA			RNA		DNA			RNA	
	D1	D2	D3	R5	R6	D4	D5	D6	R11	R12
*recA*	212.4	208.5	208.3	114.7	164.4	221.4	221.7	221.7	23.4	18.2
*gyrB*	383.1	392.1	385.6	209.5	277.1	374.8	385.7	383.8	40.6	35.5
*fusA*	788.4	800.3	794.6	434.9	594.1	764.9	782.9	774.7	201.2	183.6
*rpoB*	686.0	700.5	702.3	456.7	525.3	693.3	717.9	710.7	205.6	187.2
*infB*	356.8	359.2	359.3	229.5	298.0	345.6	376.5	368.0	63.0	50.4
*atpD*	297.5	296.5	298.0	222.9	263.9	340.7	347.9	339.5	57.3	48.1

*Samples were sequenced using Illumnia HiSeq 2500 technology, and all values were normalized for total read counts to Reads per Million (RPM). DNA samples were sequenced in triplicate (D1–D3, and D4–D6), and RNA samples were sequenced in duplicate (R5–R6, and R11–R12). The genes were identified in MG-RAST using the following annotations: *recA* (RecA protein), *gyrB* (DNA gyrase subunit B), *fusA* (Translation elongation factor G), *rpoB* (DNA-directed RNA polymerase beta subunit), *infB* (Translation initiation factor 2), and *atpD* (ATP synthase beta chain).*

## Discussion

### Standardized Workflow vs. Specific Methods

In our search to identify and overcome key problematic steps when extracting DNA/RNA from inhibitor-rich soil samples, we found that commercially available nucleic acid extraction/purification kits are not always better than non-kit methods (e.g., Nicolaisen’s method). While the DNA extraction kits fared well, none of the RNA extraction kits tested worked for all our soil samples. Even the best kit tested, the PS kit, only worked for soil Å and FH, but not for soil FL (**Table 2**). Although the PS kit was able to yield usable nucleic acids, varying quantities were extracted from equal starting amounts of a single soil type (**Figure 3A**). Considering the inherent variations in the soil, methods yielding poor replication will only further complicate matters and lead to erroneous conclusions and hypotheses. Previous studies comparing multiple methods have also concluded that extraction methods may substantially affect any downstream data (Inceoglu et al., 2010; Töwe et al., 2010). As such, we once again highlight the importance of determining suitable extraction methods based on the environment of interest. This emphasizes the need for transparent, modular methods such as the one described by Lever et al. (2015), where each step can be optimized to meet the needs for a specific sample type. Similar to their conclusions, we have found that the ease to add and adjust extraction and purification procedures as required has resulted in higher DNA and RNA yields, as well as an improved quality.

We took the study by Lever et al. (2015) further, and were able to pinpoint the important steps in nucleic acid extraction for better quality and quantity of DNA and RNA yields via our systematic testing of extraction methods. Our proposed workflow (**Figure 2**) aims to remove the problems upstream, thereby circumventing downstream problems and avoiding the struggle with persistent residual gDNA or otherwise poor quality nucleic acids. In the current study, we have chosen relative ease and speed over cost, and have opted to use commercial purification kits for each purification step. But, as suggested in our data and indicated in **Figure 2**, it is not the purification kit that determines the usability of the material downstream, but the point during extraction at which the purification step takes place – as early as possible and before enzymatic processes, but without compromising RNA stability. As such, the use of similar purification kits or methods (e.g., gradient centrifugation, Sephadex columns or chromatography) would achieve the same effect, and at a reduced cost. Similarly, the core of our suggested workflow is designed for gene expression analyses, and the restriction of total sample processing time (due to short mRNA half-lives) played a big role in the creation of our proposed workflow (**Figure 2**). Thus, our workflow reflects time-limited sample processing that is incompatible with early purification procedures that require pre-optimization, such as the addition of Al_2_(SO_4_)_3_ to remove inhibitors prior to soil disruption (Peršoh et al., 2008).

### Effectiveness of the Optimized Nucleic Acid Extraction Workflow

Although there are a large number of published modular DNA and RNA co-extraction methods, many are based on the same fundamentals of (1) sample lysis, (2) phenol-chloroform purification, and (3) nucleic acid precipitation (Griffiths et al., 2000; Arbeli and Fuentes, 2007; Nicolaisen et al., 2008; Kotiaho et al., 2010; Mettel et al., 2010; Paulin et al., 2013; Lever et al., 2015). These papers mostly focused on the buffers/materials used (e.g., composition, concentration, incubation time, etc.) and generally follow the same structure. Here, we instead aimed to characterize and detail the key order of essential steps in the workflow. In particular, additional pre-DNase digestion purification steps were added to aid in better gDNA removal and higher RNA quality. In this study, our modular method changes were grounded on Nicolaisen’s et al. (2008) method because of previous work published on the same soils (Liu et al., 2010). In that study where Nicolaisen’s method was used, both the quantity and quality was unsuitable for meta-ome sequencing, and mRNA transcripts extracted from FL soils were undetectable by qPCR, despite similar incubation conditions to those in this study (Liu et al., 2010). Using the optimized method detailed in this paper, at least double the amount of DNA and RNA was co-extracted from the same soils – Liu et al. (2010) only managed to obtain 16.1–26.4 μg DNA g^-1^ soil (ww) and 2.3–7.2 μg RNA g^-1^ soil (ww). Additionally, *nosZ* transcripts that were previously only quantifiable in soil FH (3-6 × 10^5^ copies g^-1^ soil, ww) but completely undetectable in soil FL (Liu et al., 2010), were now detectable in both soil FH and FL (see Optimized and Transparent Method for Non-kit Based Extraction).

One plausible reason behind this novel detection of *nosZ* transcripts in soil FL, could be that the higher extraction efficiency of the optimized method provided a “deeper” transcript profile. The nucleic acid yield of the optimized method presented here was ≈ 10 times that of the unmodified Nicolaisen’s method (Liu et al., 2010), and corresponded with a nearly 10-fold increase in *nosZ* transcript detection in soil FH. However, when the transcript numbers in soil FL yielded by the optimized method (1 × 10^5^ copies g^-1^ soil, ww) are adjusted to correspond with a 10 times lower efficiency (thus 1 × 10^4^ copies g^-1^ soil, ww), it is still well above than the detection limit of 8.4 × 10^3^ copies g^-1^ soil (ww) of Liu et al. (2010). Since sub-optimal extraction procedures are known to result in unusable downstream products due to persistent inhibition even after additional downstream purification processes (Cullen and Hirsch, 1998; LaMontagne et al., 2002), it is thus more likely that the quality of the isolated mRNA has improved sufficiently for *nosZ* transcript detection in soil FL. Furthermore, while the quality and quantity of RNA from soil FL yielded by Nicolaisen’s method was previously too poor for sequencing (Liu et al., 2010), the RNA yielded by the optimized method in this study from both soils were successfully sequenced and annotated (see Results **Table 4**, and Supplementary Table S3; Supplementary Figures S6 and S7). This marked improvement from undetectable mRNA, to the now successful sequencing of both metagenome and metatranscriptome using the same soils, shows that the optimized workflow greatly increased nucleic acid extraction quantity and quality.

### Enzymes, Inhibitors, and Purification

As of now, there is no existing method that can accurately determine and quantify the presence of all co-extracted enzyme inhibitors, partly due to the unknown composition of inhibitors. Their presence is instead seen through their interference with enzyme activity, affecting nucleic acid transforming processes including amplification, DNase digestion and reverse transcription. A common solution when faced with co-extracted inhibitors is to dilute the sample, reducing the degree of inhibition (Paulin et al., 2013). However, while a partially inhibited DNA amplification reaction (PCR or qPCR) may still yield usable data, using partially DNase digested RNA extracts with residual gDNA would render any RNA analysis biased and useless. Thus, since it is impossible to calculate the inhibitor-tolerance limit of all enzymatic processes (and enzyme types), it is safer and more effective to focus on purifying nucleic acids than to hope that dilution would reduce the inhibitor effect.

During our purification kit trials, we found that the sequence of steps during nucleic acid extraction is more important than the type of kit or enzyme used. We performed extensive trials using different purification kits at different stages of the extraction procedure, using only the extracts from our revised Nicolaisen’s method (commercial extraction kits had rigid procedural structures and the reagents involved were of unknown nature). We hypothesized that many commercial extraction kits failed to yield gDNA-free RNA from the inhibitor-rich soil FL, because DNase is often applied to the primary TNA extract (Extract I) before purification. The aforementioned use of the OPIR kit to purify primary TNA extracts prior to all enzymatic processes was the major breakthrough in the optimization and simplification of the extraction process. By using a specialized method to remove inhibitory compounds prior to DNase digestion, digestion efficiencies were greatly improved and the procedure was shortened significantly. In contrast, the relatively common practice of attempting to remove gDNA without purification via prolonged incubations at non-ideal RNA preservation temperatures potentially compromised the extracted RNA. Thus, it is our recommendation to purify samples prior to the digestion of gDNA to ensure maximal efficiency and speed.

If commercial kits are used for purification prior to DNase digestion, two important factors must be considered: (1) Whether or not the purification kit is RNase-free, and (2) The maximum nucleic acid holding capacity of the kit, especially for column-based purification kits. Unfortunately, DNA purification kits have higher load capacities but are not always RNase-free (e.g., gDCC), and the load capacities of the RNA purification kit columns tested were too low to capture all extracted nucleic acids (e.g., RCC). Using these potentially RNase-contaminated DNA purification kits could result in RNA digestion, whereas the RNA kits would be severely overloaded by DNA from the TNA sample. On the other hand, our kit trials revealed that the dedicated RNA purification kits are more capable of removing inhibitors than the TNA purification kit, and their use to remove residual inhibitors prior to reverse transcription was irreplaceable. Hence, while it is critical for TNA extracts (Extract II) to be purified prior to digestion, it is also essential to purify the digested extracts (Extract IV) with dedicated RNA kits to obtain high quality RNA extracts.

### Assessing DNase Digestion for RNA Purification

Using our optimized extraction and purification method, both DNA and RNA fractions were used as templates in qPCR reactions with primers targeting the 16S rRNA gene to determine the quantity and amplifiability of gDNA (**Figure 3**). There was no correlation between the quantity of residual gDNA and the starting gDNA quantities (*R*^2^ = 0.0189). The reason behind this is unclear, but uneven spread of inhibitors creates non-uniform DNase digestion of otherwise identical samples. The presence of samples with residual gDNA alongside those with no amplifiable gDNA highlights the importance of checking all samples for the presence of DNA and not only “representative samples.” Such use of “representative samples” to extrapolate the lack of contaminating residual gDNA in all RNA samples may potentially introduce severe biases with respect to the quantification and sequencing of mRNA.

A quick search of the literature using the PubMed search engine and the keywords “RNA,” “qPCR or PCR” and “transcript*” revealed a surprisingly large proportion of publications that failed to indicate or demonstrate that their RNA extracts are DNA-free. Our criteria for clear demonstration is, ideally, the use of quantification methods such as qPCR. However, we accepted the use of non-quantitative amplification analysis as a minimum indication. The analysis of unamplified nucleic acid material by electrophoresis (agarose or digital gels) or Nanodrop/Qubit quantification, was not considered sufficient evidence of samples free of amplifiable gDNA because neither is sufficiently sensitive to detect trace quantities of gDNA. Among papers published in Applied and Environmental Microbiology in 2012, 2013, and 2014, only 36, 31, and 13% clearly indicated the lack of gDNA in their RNA extracts according to our definition. This problem is not isolated to one journal, as papers published in 2014 in ISME Journal showed a similar trend, with only 37% of papers clearly addressing the residual gDNA question in RNA extracts. While more papers published in 2015 in Applied and Environmental Microbiology (47%) clearly indicated DNA-free RNA samples, the rest still either provided insufficient evidence, or failed to report that the samples had been quality-controlled prior to further downstream analysis.

While on the surface such quick assessments of gDNA removal appear beneficial, allowing a rapid analysis of the integrity of different nucleic acid fractions (as seen in Supplementary Figures S2 and S3), this creates a false impression of quality control. Low quantities of residual gDNA can still be quantifiable using qPCR in RNA samples, but may not be detectable on an agarose gel as a genomic smear even when using sensitive nucleic acid stains such as GelRed (Biotium) or peqGREEN (Peqlab; data not shown). Our qPCR analysis revealed the presence of substantial quantities of gDNA (**Figure 3**), even though gel visualization (not shown) failed to reveal the presence of gDNA in the purified RNA fraction. Additionally, using either spectrofluoro- or spectrophotometric methods to quantify residual gDNA relies heavily on exceeding minimum detection limits, as well as the assumption that the fluorophores have not been otherwise inhibited (Bachoon et al., 2001; Zipper et al., 2003; Sidstedt et al., 2015), neither of which can be easily presumed where environmental samples are concerned. Thus, we strongly recommend the use of quantitative methods such as qPCR (or amplification procedures at the very least, to amplify the signal from trace gDNA molecules) to definitively determine the efficiency of DNase digestion reactions to avoid overestimations of active microbial communities in soil due to the presence of contaminating gDNA.

## Concluding Remarks

As is known from other studies and indicated in **Table 2**, kits and methods that work well for one soil may not perform similarly for another soil type. Our results highlight how soil types with different properties can affect the quality of nucleic acids extracted via identical methods. This disparity likely arises from the unique inhibitor profiles of each soil type, which in turn interfere with the various nucleic acid transforming enzymes to different extents. As such, it is important to thoroughly purify nucleic acids as much as possible prior to any enzymatic process, including but not restricted to DNase digestion, reverse transcription and amplification. Such purification results in more efficient and effective DNase digestion, reducing incubation times and consequently reducing RNA placement at non-optimal temperatures. However, even with multiple purification techniques, DNase digestion is not always a uniform process (especially with inhibitor-rich soil extracts), and the residual gDNA may vary between samples and replicates. Thus, we strongly recommend the examination of all samples for residual gDNA and not only “representative samples.” Furthermore, we propose the use of the more sensitive qPCR method as an indicator of residual gDNA, rather than less sensitive methods such as electrophoretic analysis of unamplified nucleic acid extracts.

## Author Contributions

All authors contributed to the planning of the work and the revision of the manuscript. In addition, NL and CR performed the experimental work detailed in this paper. NL performed data analysis and the drafting of the manuscript.

## Conflict of Interest Statement

The authors declare that the research was conducted in the absence of any commercial or financial relationships that could be construed as a potential conflict of interest.
