# Supplementary material for: Transparent DNA/RNA Co-extraction Workflow Protocol Suitable for Inhibitor-Rich Environmental Samples That Focuses on Complete DNA Removal for Transcriptomic Analyses
Source: Front Microbiol. 2016 Oct 18;7:1588. doi: 10.3389/fmicb.2016.01588 (PMC5067521; doi:10.3389/fmicb.2016.01588)
Supplement: Supplementary file 1 [file Data_Sheet_1.pdf]

Supplementary Material

Transparent DNA/RNA co-extraction workflow protocol suitable for inhibitor-rich environmental samples that focuses on complete DNA removal for transcriptomic analyses

Natalie Y.N. Lim*, Constance A. Roco & Åsa Frostegård

*** Correspondence:** Natalie Y.N. Lim: natalie.lim@nmbu.no

1 Supplementary Data

1.1 The effectiveness of dedicated nucleic acid extraction kits

To create a baseline comparison with other studies and the commercial standard, nucleic acids were extracted from the high (FH) and low (FL) pH peat soils using several kits. As stated in the Materials and Methods, all nucleic acid quantities were measured with Qubit, whereas quality assessment was performed with NanoDrop (numerical values from absorbance ratios) and/or agarose gel visualization of the degree of shearing. None of the extraction kits tested was able to provide both usable DNA and mRNA from both soil types. Additionally, the kits varied in the overall quality and quantity of extracted DNA and RNA, for both dedicated DNA extraction kits and kits that co-extracted DNA and RNA (see Table S2). Aside from the already unacceptable $A_{260/280}$ ratios, the kits also yielded very poor $A_{260/230}$ ratios: ranging from 0.26-1.66 before additional purification, and improving to 1.77-2.14 after purification (primary extracts under 1.0 were considered failures and were not purified).

The gDNA extracted from these kits also differed in the degree of shearing (ranging from approximately 2 to 8 kb and represented by the degree of smearing when run on an agarose gel; which may be potentially important for metagenomic studies, see Figure S3). Notably, despite its intention to extract only RNA by keeping DNA bound to the column, a high molecular weight band on the gel was still clearly visible in the RNA extract from the PS kit, indicating the co-elution of DNA with RNA (see Figure S3).

All the extraction kits performed relatively well with FH soil – some required further purification with a purification kit to achieve stronger amplification of the 16S rRNA or *nosZ* genes, but some amplification was generally achieved even without (see Figure S4). However, when tested on FL, most extraction kits could not yield amplifiable DNA without the aid of further purification (see Figure S4). Interestingly, some kits performed so poorly that the obtained extract from FL remained unusable after subsequent purification with one or more of the purification kits. This is likely due to high quantities of co-extracted inhibitors, and is in line with other studies showing the failure of downstream purification techniques to tackle extremely high levels of humic acid contamination (Cullen and Hirsch, 1998; LaMontagne et al., 2002; Young et al., 2014).

1.2 Optimized lysis and precipitation

Since none of the extraction kits yielded usable nucleic acids from all three soils, we chose to modify a commonly used non-kit method because it gave us more freedom to optimize the individual procedures involved in the extraction process. Nucleic acids were extracted from

high (FH) and low (FL) pH peat soils using Nicolaisen's method, but different lysis procedures, buffers and precipitants were tested. The best lysis was achieved with the three sizes of glass beads, two cycles of lysis (45 s each) using the FastPrep-24 Instrument, and using CTAB extraction buffer and phenol (both buffered to pH 8.0). There was no significant difference when garnet (14.89 ± 3.57 µg DNA g^{-1} soil) or glass (16.04 ± 3.75 µg DNA g^{-1} soil) beads were used ($p > 0.1$), but the quantity extracted increased when three sizes of beads was used (4.27 µg DNA g^{-1} soil) instead of one size (3.17 µg DNA g^{-1} soil). Although there was no significant difference in nucleic acid quantity when using the FastPrep-24 Instrument compared to the vortex ($p > 0.1$), the FastPrep-24 Instrument was used in further experiments for reasons of comparability with existing literature, due to its widespread use (Griffiths et al., 2000; Kotiaho et al., 2010; Mettel et al., 2010; Nicolaisen et al., 2008). The extent of gDNA shearing varied directly with the number of lysis cycles, where one lysis cycle yielded the largest fragments of gDNA (see Figure S5). However, there was no such correlation with the amplifiability (see Figure S5) or quantity of nucleic acid material where bead beating twice yielded the most nucleic acid material (1×, 2× and 3× bead beating generated 4.26 ± 1.67 µg DNA g^{-1} soil, 9.51 ± 1.88 µg DNA g^{-1} soil, and 2.93 ± 2.45 µg DNA g^{-1} soil, respectively). Thus, two cycles of lysis was chosen because it yielded the highest quantity of nucleic acid material obtained, and the gDNA was not badly sheared. For lysis buffers, aside from the pH 8.0 buffered CTAB and phenol, all combinations involving acidic CTAB, other buffer agents, GES buffer, acidic phenol or increased buffer ionic strength either failed to extract RNA (detection limit 0.01 µg RNA g^{-1} soil) or co-extracted large quantities of inhibitors, effectively preventing all downstream processes.

In this study, we used isopropanol as a precipitant to further reduce incubation times. Not only did it require a shorter precipitation time than PEG (2 minutes versus 2 hours), precipitation with isopropanol yielded up to threefold increase of DNA over PEG. Isopropanol consistently yielded higher quantities of both DNA and RNA than PEG 6000, with little cost to nucleic acid purity – precipitation with PEG 6000 yielded 50-75 µg DNA g^{-1} soil (ww), whereas isopropanol precipitation yielded 50-150 µg DNA g^{-1} soil (ww). Thus, where applicable, isopropanol was used as the precipitant in all subsequent extractions. Although there is some contrasting opinion on the role isopropanol may play in co-precipitating or removing inhibitory compounds (Arbeli and Fuentes, 2007; Cullen and Hirsch, 1998; Hänni et al., 1995; Krsek and Wellington, 1999; LaMontagne et al., 2002), early trials in this study comparing the use of isopropanol and PEG had indicated little disadvantage in using isopropanol. Additionally, alcohols are known to provide better yields (Krsek and Wellington, 1999), and isopropanol has also previously been recommended as the precipitant of choice for its potential ability to remove polysaccharides from soil (Cullen and Hirsch, 1998).

2 Supplementary Tables

TABLE S1 | Buffer-phenol combinations tested in the present study

Buffer	Components	Buffer used	Buffer pH	Buffer ionic strength	Phenol pH
Normal [a]	5 % w/v CTAB<br>0.35 M NaCl<br>1 % w/v PVPP	Phosphate buffer	8.0	120 mM	8.0
Strong phosphate	10 % w/v CTAB<br>0.35 M NaCl<br>1 % w/v PVPP	Phosphate buffer	8.0	250 mM	8.0
Strong Tris	10 % w/v CTAB<br>0.35 M NaCl<br>1 % w/v PVPP	Tris buffer	8.0	250 mM	8.0
Acidic phenol only	10 % w/v CTAB<br>0.35 M NaCl<br>1 % w/v PVPP	Phosphate buffer	8.0	120 mM	4.0
Acidic buffer and phenol	10 % w/v CTAB<br>0.35 M NaCl<br>1 % w/v PVPP	Phosphate buffer	5.7	120 mM	4.0
GES buffer [b]	5 M guanidinium thiocyanate<br>100 mM EDTA<br>0.5 % sarcosyl	Acetate buffer	4.0	25 mM	4.0

[a] A modified phenol-chloroform extraction method as published previously by Nicolaisen and colleagues (Nicolaisen et al., 2008).

[b] GES: Guanidinium thiocyanate-EDTA-sarcosyl

TABLE S2 | Comparison of DNA/RNA extraction kits, tested on soils FH (high pH peat, pH 7.39) and FL (low pH peat, pH 3.65).

Kit		FH				FL			
		DNA		RNA		DNA		RNA	
		μg g^{-1} soil (ww)	$A_{260/280}$	μg g^{-1} soil (ww)	$A_{260/280}$	μg g^{-1} soil (ww)	$A_{260/280}$	μg g^{-1} soil (ww)	$A_{260/280}$
PowerLyzer PowerSoil DNA Isolation Kit	PL	24.5	1.79	-	-	4.02	1.56	-	-
FastDNA SPIN Kit for Soil	FDS	16.0	1.71	-	-	4.09	1.71	-	-
ZR Soil Microbe DNA MiniPrep	SM	15.4	1.76	-	-	4.82	1.68	-	-
MasterPure RNA Purification Kit	MP	88.9 (14.7)*	1.53 (1.84)*	5.54	1.71	85.1 (16.0)*	1.78 (1.84)*	3.20	1.67
PowerMicrobiome RNA Isolation	PM	19.3 (15.3)*	1.64 (1.87)*	23.4 (15.6)*	1.64 (2.57)*	15.5 (9.51)*	1.64 (1.85)*	16.45 (9.09)*	1.60 (2.75)*
RNA PowerSoil Total RNA Isolation Kit	PS	-	-	-	-	-	-	6.71	1.92
RNA PowerSoil Total RNA Isolation Kit	PS + AK	45.5	1.50	19.5 (14.6)*	1.56 (1.57)*	37.9	1.53	18.6 (13.96)*	1.61 (1.58)*

All values listed are averages of triplicate extractions.

* Kits that yielded colored extracts (a sign of very large quantities of inhibitory compounds) were further purified. The values within the parentheses are post-purification with the Genomic DNA Clean & Concentrator or RNA Clean & Concentrator – 5

TABLE S3 | Summary of MG-RAST annotated meta-omics data

Soil type	Sample type	ID	Total reads	Passed QC	Average length (bp)
FH	DNA	D1	28 674 145	97.4 %	155
FH	DNA	D2	31 420 570	97.5 %	155
FH	DNA	D3	29 448 386	97.2 %	155
FL	DNA	D4	29 142 448	97.3 %	155
FL	DNA	D5	30 690 762	97.3 %	155
FL	DNA	D6	25 949 776	96.9 %	155
FH	RNA	R1	17 902 594	81.9 %	128
FH	RNA	R2	24 855 082	97.7 %	129
FH	RNA	R3	17 767 603	98.7 %	121
FH	RNA	R4	16 441 508	97.4 %	128
FH	RNA	R5	17 993 765	99.5 %	116
FH	RNA	R6	27 809 492	98.8 %	127
FH	RNA	R7	42 039 146	95.1 %	134
FH	RNA	R8	4 030 430	95.6 %	133
FH	RNA	R9	22 104 868	96.5 %	110
FH	RNA	R10	17 735 486	98.2 %	129
FL	RNA	R11	8 466 353	97.3 %	119
FL	RNA	R12	17 111 152	97.5 %	121
FL	RNA	R13	21 605 163	98.8 %	125
FL	RNA	R14	19 321 965	97.6 %	120

Samples were sequenced with Illumina HiSeq 2500 technology. The samples were trimmed for adaptors and quality controlled to remove short sequences (< 80 bp), then submitted to MG-RAST for annotation. The annotated FH and FL soil sequences are available online in the MG-RAST database (project ID 14446, project name “Fjaler_HiSeq”).

3 Supplementary Figures

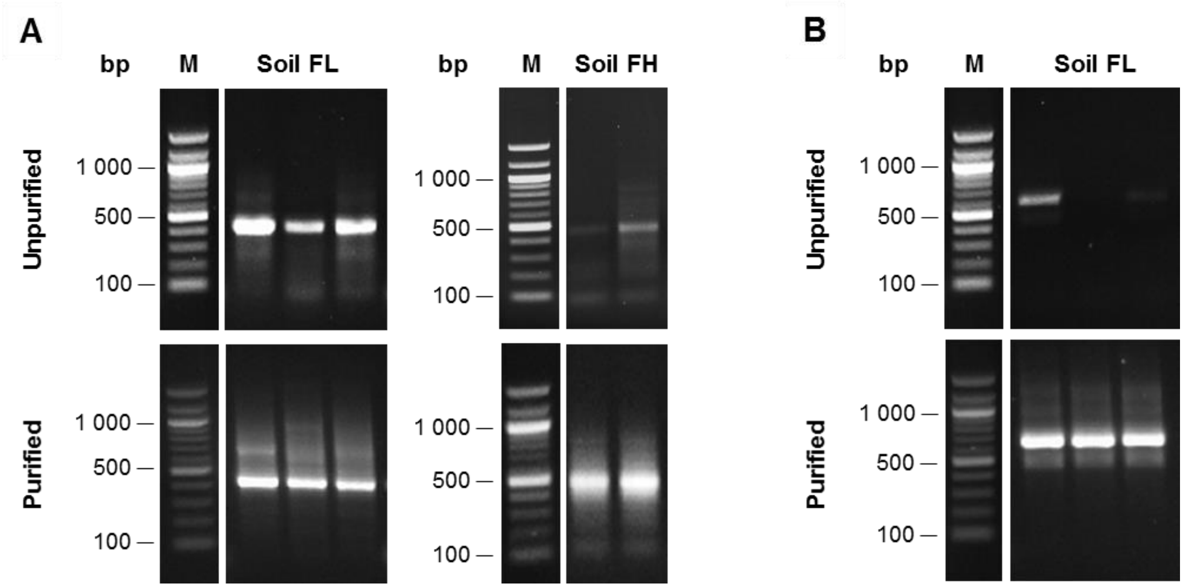

FIGURE S1 | Purifying crude DNA extracts prior to PCR gave stronger and more consistent amplification, regardless of primer used. Triplicate and duplicate samples were extracted from soils FL and FH respectively, using the unmodified Nicolaisen's method. Purified (using the Genomic DNA Clean & Concentrator kit) and unpurified DNA extracts were used in amplification reactions with primers targeting the A) *nosZ* gene (Z-F/1622R, expected amplicon size ~453 bp); or B) *narG* gene (1960f/2650r, expected amplicon size ~650 bp), and equal quantities of product were loaded onto the gels. The intensity of bands were compared by using the marker (M: 100 bp DNA ladder) as a standard of comparison across gels. The 'dimmer' marker bands in the "purified" gels reflect the intensity of the amplicons of interest, which required a shorter gel exposure period for the photograph.

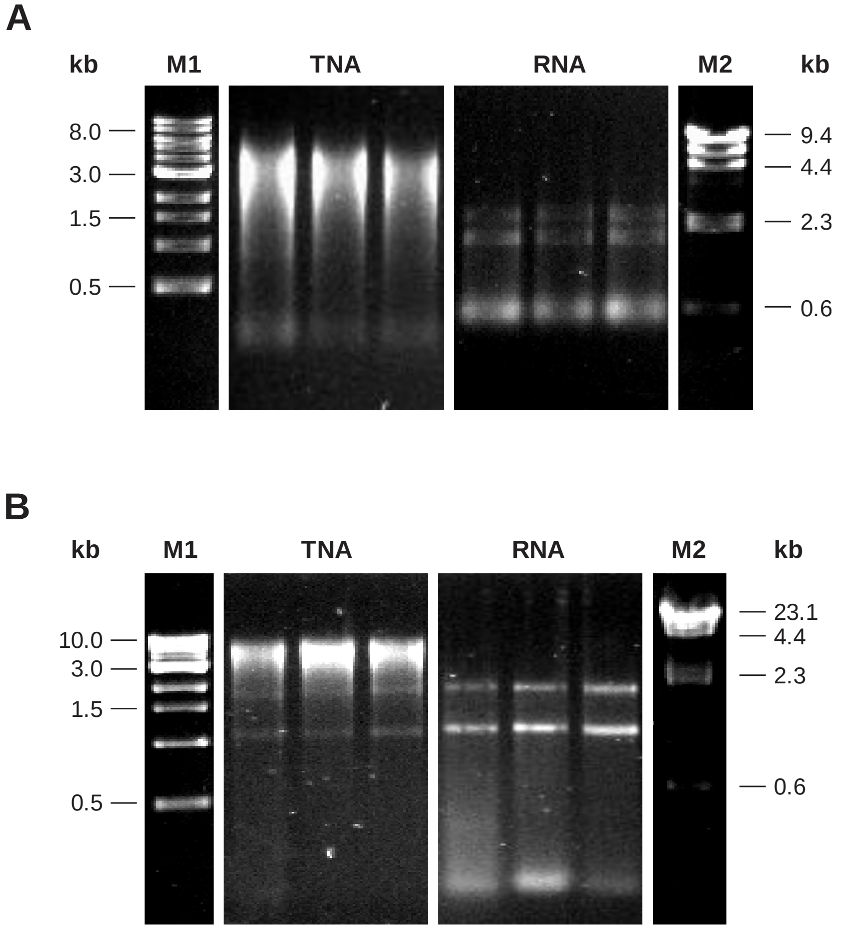

FIGURE S2 | Our simplified extraction method is capable of yielding RNA with little or no residual genomic DNA (gDNA) in the RNA fraction. Triplicate unamplified crude TNA extracts and DNase-digested RNA from soil FL using our simplified extraction method were analyzed on agarose gels to quickly assess the integrity of the nucleic acids. The gDNA smear (between 3 and 8 kb) is easily differentiated from the rRNA bands (the smaller bands under the gDNA smear) by gel electrophoresis. (A) The first gel was used to differentiate higher molecular weight fragments, to analyze the size of the gDNA smear. (B) The second gel was used to clearly separate the two smaller bands (presumed to be 23S and 16S rRNA) from the gDNA smear. The optimized purification of RNA prior to digestion retained most of the 16S and 23S rRNA despite complete digestion of gDNA (further confirmed with qPCR). Equivalent quantities of nucleic acid material (based on g^{-1} soil wet weight) was loaded into each well. Note that the gel has been spliced to remove unrelated samples, and the size of the RNA bands cannot be compared to the DNA markers used. M1: 1 kb DNA ladder. M2: λ DNA-HindIII marker.

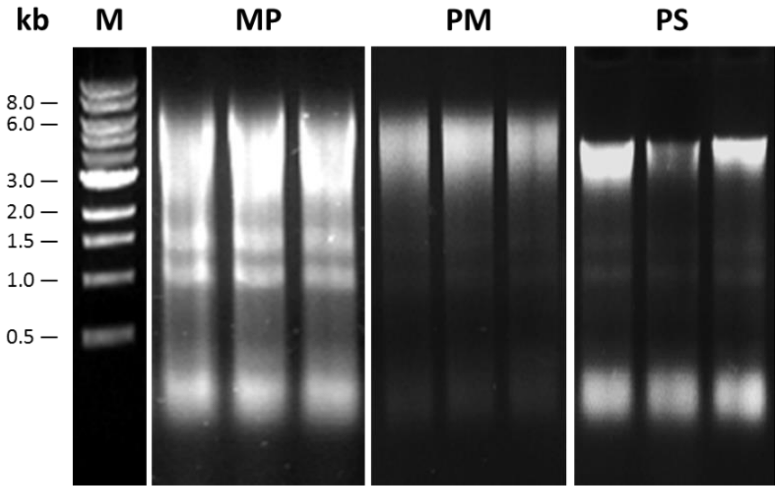

FIGURE S3 | Triplicate crude total nucleic acid (TNA) extracts was analyzed on a 1 % agarose gel to assess the integrity of DNA and RNA extracted from soil FL. The kits were used to extract TNA from three replicate soil samples. The gDNA smear (between 3 and 8 kb) is easily differentiated from the rRNA bands (the smaller bands under the gDNA smear) by gel electrophoresis. Equivalent quantities of extract (based on g^{-1} soil wet weight) were loaded into each well, showing clearly that some kits yield very dilute nucleic acid material. The PowerSoil RNA kit was supposed to elute only RNA, but there is obvious evidence of gDNA in all replicates, and very weak RNA bands are present, representative of the low amounts of RNA that is extracted. MP: MasterPure RNA Purification Kit (Epicentre Biotechnologies), PM: PowerMicrobiome RNA Isolation Kit, PS: RNA PowerSoil Total RNA Isolation Kit (both from MO BIO Laboratories). Note that the gels have been spliced for purposes of comparison. M: 1 kb DNA ladder.

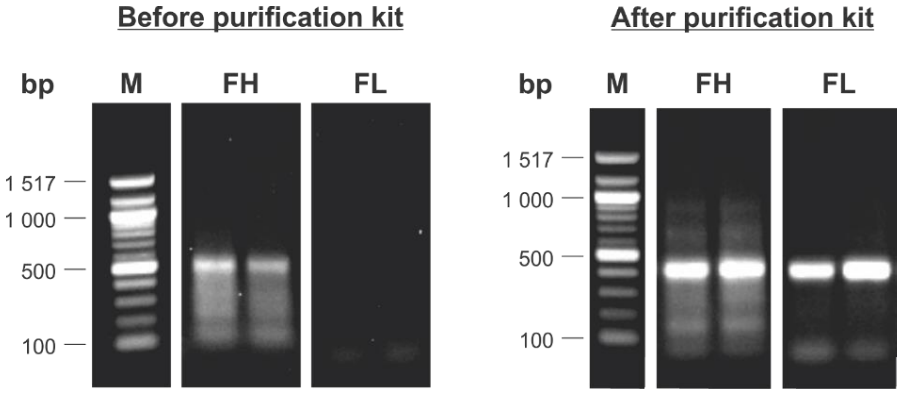

FIGURE S4 | Duplicate primary DNA extracts (Extract I, as seen in FIGURE 2) obtained with extraction kits from soil FH were amplifiable, but not from soil FL. Purification with the Genomic DNA Clean & Concentrator kit (gDCC) resulted in successful DNA amplification of the FL extract, and brighter amplicon bands from the FH extract (as seen by brighter non-specific amplicons greater than the expected size). The example above shows DNA extracts from soils FH and FL obtained using the PowerLyzer PowerSoil DNA Isolation Kit (PL), amplified with primers Z-F and 1622R targeting the *nosZ* gene, with an expected amplicon size of approximately 450 bp. Equal quantities of product were loaded onto the gels. The same trend was observed with other extraction kits. M: 100 bp DNA ladder.

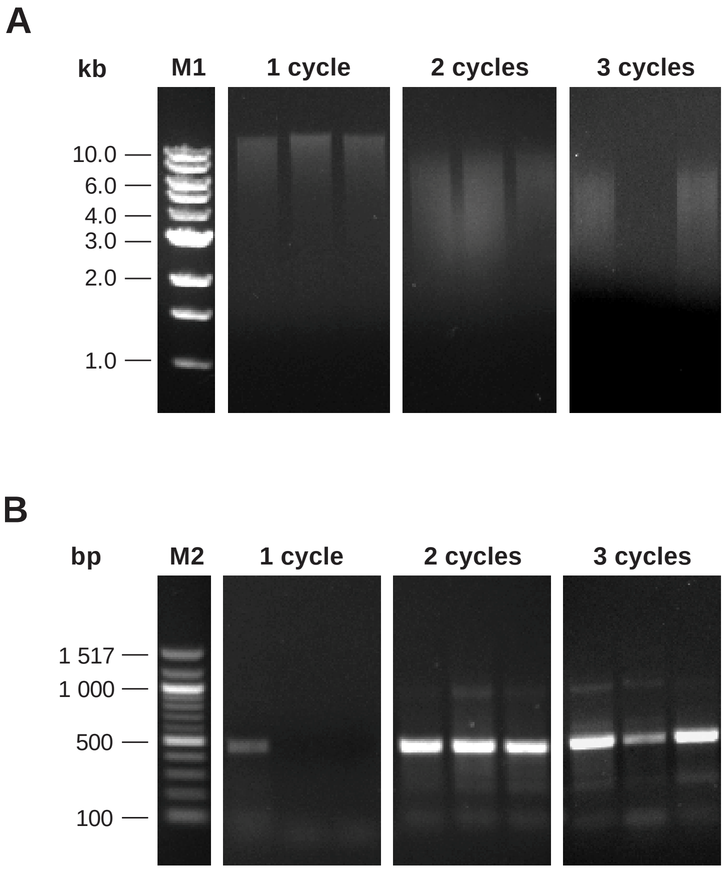

FIGURE S5 | The number of mechanical lysis cycles (via bead beating) affects both genomic DNA (gDNA) shear and amplifiability. Triplicate samples were extracted using the otherwise unmodified Nicolaisen's method and resulting nucleic acids were purified with the Genomic DNA Clean & Concentrator kit (gDCC). (A) The gDNA smear size decreased when samples were put through more than one cycle of bead beating, but there is no visible difference between two or three cycles of bead beating. The faint genomic smear is caused by low extraction efficiencies of a non-optimized method. (B) One cycle of bead beating did not yield amplifiable DNA, and three cycles of bead beating yielded more unspecific amplicons (as judged by stronger bands of the wrong fragment size). PCR was performed with primers Z-F and 1622R targeting the *nosZ* gene, with an expected amplicon size of approximately 450 bp. Equal quantities of product were loaded onto the gels. M1: 1 kb DNA ladder. M2: 100 bp DNA ladder.

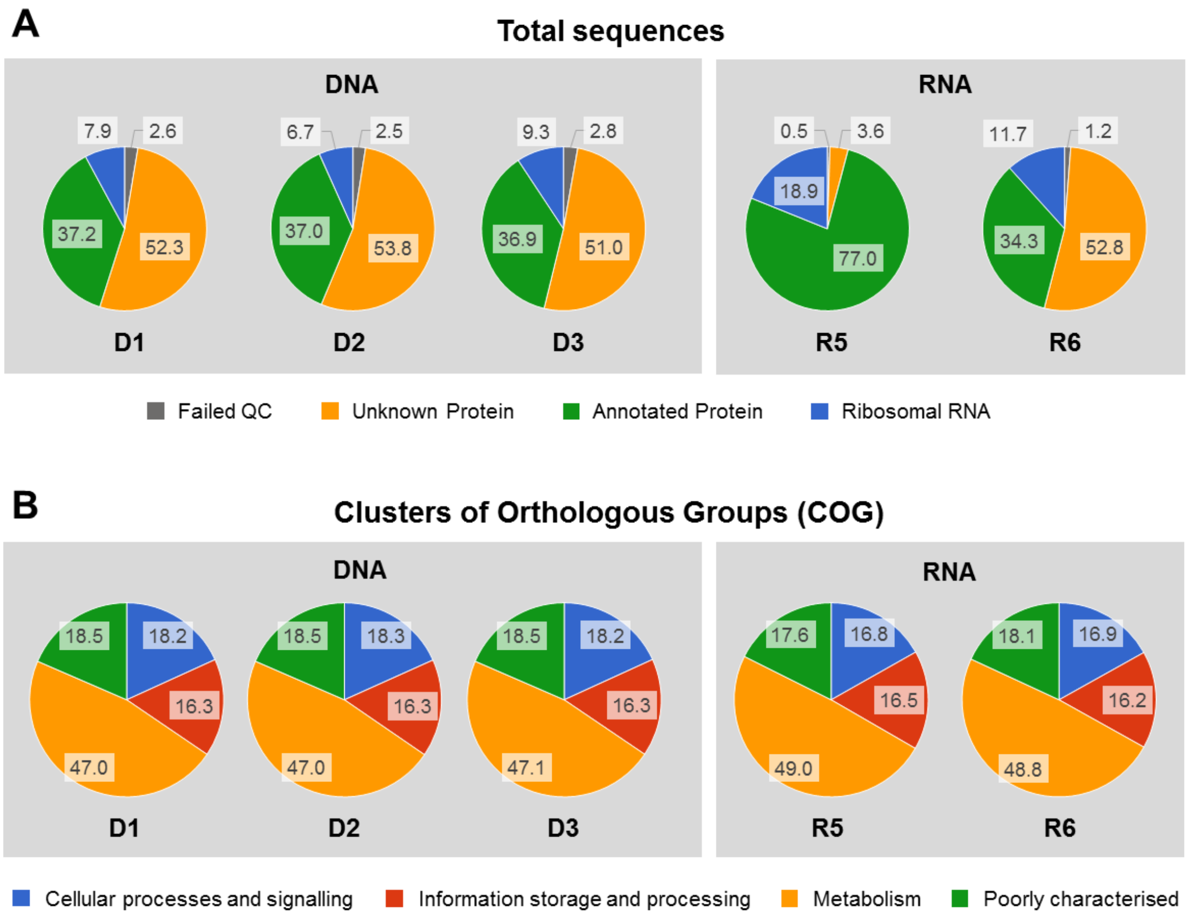

FIGURE S6 | Graphical breakdown of sequenced DNA and RNA samples extracted from soil FH (pH 6.80). DNA and RNA samples were sequenced in triplicate (D1, D2 and D3) and duplicate (R5 and R6), respectively. MG-RAST-annotated profiles were generated using A) Total sequences, and B) Clusters of Orthologous Groups (COG). The COG profile was generated from the green "Annotated Protein" segment of Total Sequences (A). Despite a higher proportion of "unknown protein" sequences in R6, the COG profile indicates good co-extraction replication for both DNA and RNA samples using the optimized method.

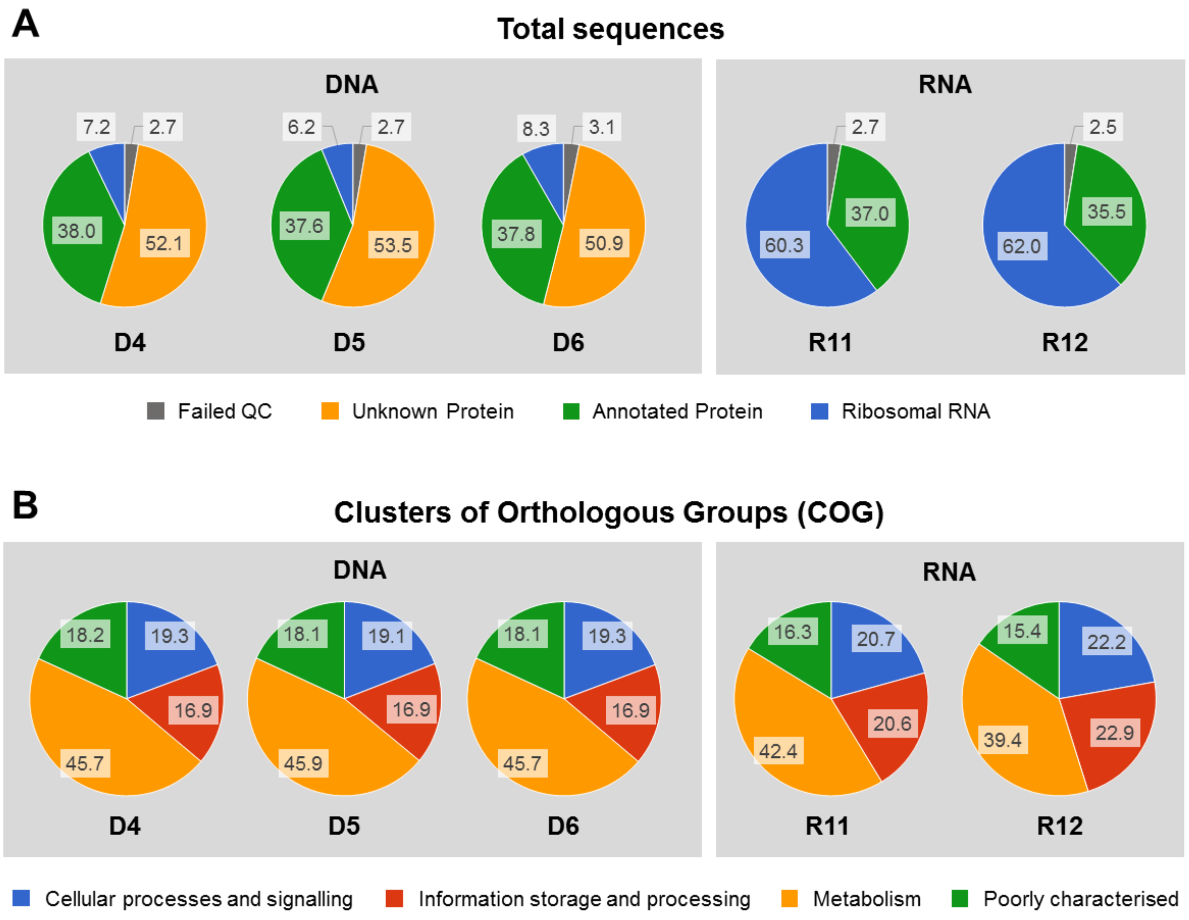

FIGURE S7 | Graphical breakdown of sequenced DNA and RNA samples extracted from soil FL (pH 3.80). DNA and RNA samples were sequenced in triplicate (D4, D5 and D6) and duplicate (R11 and R12), respectively. MG-RAST-annotated profiles were generated using A) Total sequences, and B) Clusters of Orthologous Groups (COG). The COG profile was generated from the green "Annotated Protein" segment of Total Sequences (A). Both Total Sequence and COG profiles indicate good co-extraction replication for both DNA and RNA samples using the optimized method.

4 References

Arbeli, Z., and Fuentes, C. L. (2007). Improved purification and PCR amplification of DNA from environmental samples. *FEMS Microbiol. Lett.* 272, 269–75. doi:10.1111/j.1574-6968.2007.00764.x.

Cullen, D. W., and Hirsch, P. R. (1998). Simple and rapid method for direct extraction of microbial DNA from soil for PCR. *Soil Biol. Biochem.* 30, 983–993. doi:10.1016/S0038-0717(98)00001-7.

Griffiths, R. I., Whiteley, A. S., O'Donnell, A. G., and Bailey, M. J. (2000). Rapid Method for Coextraction of DNA and RNA from Natural Environments for Analysis of Ribosomal DNA- and rRNA-Based Microbial Community Composition. *Appl. Environ. Microbiol.* 66, 5488–5491. doi:10.1128/aem.66.12.5488-5491.2000.

Hänni, C., Brousseau, T., Laudet, V., and Stehelin, D. (1995). Isopropanol precipitation removes PCR inhibitors from ancient bone extracts. *Nucleic Acids Res.* 23, 881–882. doi:10.1093/nar/23.5.881.

Kotiaho, M., Fritze, H., Merilä, P., Juottonen, H., Leppälä, M., Laine, J., et al. (2010). Methanogen activity in relation to water table level in two boreal fens. *Biol. Fertil. Soils* 46, 567–575. doi:10.1007/s00374-010-0461-0.

Krsek, M., and Wellington, E. M. H. (1999). Comparison of different methods for the isolation and purification of total community DNA from soil. *J. Microbiol. Methods* 39, 1–16. doi:10.1016/S0167-7012(99)00093-7.

LaMontagne, M. G., Michel, F. C., Holden, P. A., and Reddy, C. A. (2002). Evaluation of extraction and purification methods for obtaining PCR-amplifiable DNA from compost for microbial community analysis. *J. Microbiol. Methods* 49, 255–264. doi:10.1016/S0167-7012(01)00377-3.

Mettel, C., Kim, Y., Shrestha, P. M., and Liesack, W. (2010). Extraction of mRNA from Soil. *Appl. Environ. Microbiol.* 76, 5995–6000. doi:10.1128/aem.03047-09.

Nicolaisen, M. H., Baelum, J., Jacobsen, C. S., and Sørensen, J. (2008). Transcription dynamics of the functional tfdA gene during MCPA herbicide degradation by Cupriavidus necator AEO106 (pRO101) in agricultural soil. *Environ. Microbiol.* 10, 571–9. doi:10.1111/j.1462-2920.2007.01476.x.

Young, J. M., Rawlence, N. J., Weyrich, L. S., and Cooper, A. (2014). Limitations and recommendations for successful DNA extraction from forensic soil samples: a review. *Sci. Justice* 54, 238–44. doi:10.1016/j.scijus.2014.02.006.
